# Multi-atlas segmentation with joint label fusion and corrective learning—an open source implementation

**DOI:** 10.3389/fninf.2013.00027

**Published:** 2013-11-22

**Authors:** Hongzhi Wang, Paul A. Yushkevich

**Affiliations:** Department of Radiology, PICSL, Perelman School of Medicine at the University of PennsylvaniaPhiladelphia, PA, USA

**Keywords:** multi-atlas label fusion, joint label fusion, corrective learning, Insight-Toolkit, open source implementation

## Abstract

Label fusion based multi-atlas segmentation has proven to be one of the most competitive techniques for medical image segmentation. This technique transfers segmentations from expert-labeled images, called atlases, to a novel image using deformable image registration. Errors produced by label transfer are further reduced by label fusion that combines the results produced by all atlases into a consensus solution. Among the proposed label fusion strategies, weighted voting with spatially varying weight distributions derived from atlas-target intensity similarity is a simple and highly effective label fusion technique. However, one limitation of most weighted voting methods is that the weights are computed independently for each atlas, without taking into account the fact that different atlases may produce similar label errors. To address this problem, we recently developed the joint label fusion technique and the corrective learning technique, which won the first place of the 2012 MICCAI Multi-Atlas Labeling Challenge and was one of the top performers in 2013 MICCAI Segmentation: Algorithms, Theory and Applications (SATA) challenge. To make our techniques more accessible to the scientific research community, we describe an Insight-Toolkit based open source implementation of our label fusion methods. Our implementation extends our methods to work with multi-modality imaging data and is more suitable for segmentation problems with multiple labels. We demonstrate the usage of our tools through applying them to the 2012 MICCAI Multi-Atlas Labeling Challenge brain image dataset and the 2013 SATA challenge canine leg image dataset. We report the best results on these two datasets so far.

## 1. Introduction

Image segmentation is often necessary for quantitative medical image analysis. In most applications, manual segmentation labeled by human expert is treated as the gold standard. However, due to the high labor intensive nature of manual segmentation and its poor reproducibility, it is often desirable to have accurate automatic segmentation techniques to replace manual segmentation.

As an intuitive solution for applying manually labeled images to segment novel images, atlas-based segmentation (Rohlfing et al., 2005) has been widely applied in medical image analysis. This technique applies example-based knowledge representation, where the knowledge for segmenting a structure of interest is represented by a pre-labeled image, called an atlas. Through establishing one-to-one correspondence between a target novel image and an atlas image by image-based deformable registration, the segmentation label can be transferred to the target image from the atlas.

Segmentation errors produced by atlas-based segmentation are mostly due to registration errors. One effective way to reduce such errors is through employing multiple atlases. When multiple atlases are available, each atlas produces one candidate segmentation for the target image. Under the assumption that segmentation errors produced by different atlases are not identical, it is often feasible to derive more accurate solutions by label fusion. Since the example-based knowledge representation and registration-based knowledge transfer scheme can be effectively applied in many biomedical imaging problems, label fusion based multi-atlas segmentation has produced impressive automatic segmentation performance for many applications (Rohlfing et al., 2004; Isgum et al., 2009; Collins and Pruessner, 2010; Asman and Landman, 2012; Wang et al., 2013a). For some most studied brain image segmentation problems, such as hippocampus segmentation (Wang et al., 2011) and hippocampal subfield segmentation (Yushkevich et al., 2010), automatic segmentation performance produced by multi-atlas label fusion has reached the level of inter-rater reliability.

Weighted voting with spatially varying weight distributions derived from atlas-target intensity similarity is a simple and highly effective label fusion technique. However, most weighted voting methods compute voting weights independently for each atlas, without taking into account the fact that different atlases may produce similar label errors. To address this problem, we developed the joint label fusion technique (Wang et al., 2013b) and the corrective learning technique (Wang et al., 2011). To make our techniques more accessible to the scientific research community, we describe an Insight-Toolkit based implementation of our label fusion methods. Our work has the following novel contributions. First, we extend our label fusion techniques to work with multi-modality imaging data and with user designed features. Second, we simplify the usage and improve the efficiency of the corrective learning technique to make it more suitable for segmentation problems with multiple labels. Both theoretical and implementation issues are discussed in detail. We demonstrate the usage of our software through two applications: brain magnetic resonance image (MRI) segmentation using the data from the 2012 MICCAI Multi-Atlas Labeling Challenge (Landman and Warfield, 2012) and canine leg muscle segmentation using the data from 2013 SATA challenge. We report the best segmentation results on these two datasets so far.

## 2. Materials and methods

### 2.1. Method overview

#### 2.1.1. Multi-atlas segmentation with joint label fusion

Let *T*_*F*_ be a target image to be segmented and *A*^1^ = (*A*^1^_*F*_, *A*^1^_*S*_), …, *A*^*n*^ = (*A*^*n*^_*F*_, *A*^*n*^_*S*_) be *n* atlases, warped to the space of the target image by deformable registration. *A*^*i*^_*F*_ and *A*^*i*^_*S*_ denote the *i*_*th*_ warped atlas image and manual segmentation. Joint label fusion is a weighted voting based label fusion technique.

Weighted voting is a simple yet highly effective approach for label fusion. For instance, majority voting (Rohlfing et al., 2005; Heckemann et al., 2006) applies equal weights to every atlas and consistently outperforms single atlas-based segmentation. Among weighted voting approaches, similarity-weighted voting strategies with spatially varying weight distributions have been particularly successful (Artaechevarria et al., 2009; Isgum et al., 2009; Sabuncu et al., 2010; Yushkevich et al., 2010; Wang et al., 2013b). The consensus votes received by label *l* are:

(1) $$\hat{p}\left(l|x,T_{F}\right)=\sum_{i=1}^{n}w_{x}^{i}p\left(l|x,A^{i}\right)$$

where $\hat{p}\left(l|x,T_{F}\right)$ is the estimated probability of label *l* for the target image at location *x*. *p*(*l*|*x*, *A*^*i*^) is the probability that *A*^*i*^ votes for label *l* at *x*, with ∑_*l* ∈ {1, …, *L*}_*p*(*l*|*x*, *A*^*i*^) = 1. *L* is the total number of labels. Note that for deterministic atlases that have one unique label for every location, *p*(*l*|*x, A*^*i*^) degenerates into an indicator function, i.e., *p*(*l*|*x, T*_*F*_) = *I*(*T*_*S*_(*x*) = *l*) and *p*(*l*|*x, A*^*i*^) = *I*(*A*^*i*^_*S*_(*x*) = *l*), where *T*_*S*_ is the unknown segmentation for the target image. *w*^*i*^_*x*_ is the voting weight for the *i*_*th*_ atlas, with $\sum_{i=1}^{n}w_{x}^{i}=1$.

***2.1.1.1. The joint label fusion model Wang et al., 2013b***. For deterministic models, we model segmentation errors produced by each warped atlas as δ^*i*^(*x*) = *I*(*T*_*S*_(*x*) = *l*) − *I*(*A*^*i*^_*S*_(*x*) = *l*). Hence, δ^*i*^(*x*) ∈ {−1, 0, 1} is the observed label difference. The correlation between any two atlases in producing segmentation errors at location *x* are captured by a dependency matrix *M*_*x*_, with *M*_*x*_(*i, j*) = *p*(δ^*i*^(*x*)δ^*j*^(*x*) = 1 | *T*_*F*_, *A*^*i*^_*F*_, *A*^*j*^_*F*_) measuring the probability that atlas *i* and *j* produce the same label error for the target image. The expected label difference between the consensus solution obtained from weighted voting and the target segmentation is:

(2) $$\begin{array}{l} E_{\delta^{1}(x),\ldots ,\delta^{n}(x)}[{\left(I(T_{S}(x)=l)-\sum_{i=1}^{n}w_{x}^{i}I\left(A_{S}^{i}(x)=l\right)\right)}^{\text{​}2}\text{​}|\\ \;T_{F},A_{F}^{1},\ldots ,A_{F}^{n}]=w_{x}^{t}M_{x}w_{x} \end{array}$$

where *t* stands for transpose. To minimize the expected label difference, the optimal voting weights can be solved by $w_{x}=\frac{M_{x}^{-1}1_{n}}{1_{n}^{t}M_{x}^{-1}1_{n}}$, where 1_*n*_ = [1; 1;…;1] is a vector of size *n*. To avoid inverting an ill-conditioned matrix, we always add an identity matrix weighted by a small positive number α to *M*_*x*_.

*The key difference between joint label fusion and other label fusion methods is that it explicitly considers correlations among atlases, i.e., the dependence matrix, into voting weight assignment to reduce bias in the atlas set*. In the extreme example, if one of the atlases in the atlas set is replicated multiple times, the combined weight assigned to all replicates of the atlas would be the same as when the atlas is included only once. This is in contrast to earlier weighted voting label fusion methods (Artaechevarria et al., 2009; Sabuncu et al., 2010), in which the weight assigned to the replicated atlas increases with the number of replicates. More generally, the weights assigned by joint label fusion to anatomical patterns in the atlases are not biased by the prevalence of those patterns in the atlas set.

***2.1.1.2. Estimation of the pairwise atlas dependency matrix *M*_*x*_.*** Since the segmentation of the target image is unknown, we apply an image similarity based model over local image patches to estimate *M*_*x*_ as follows:

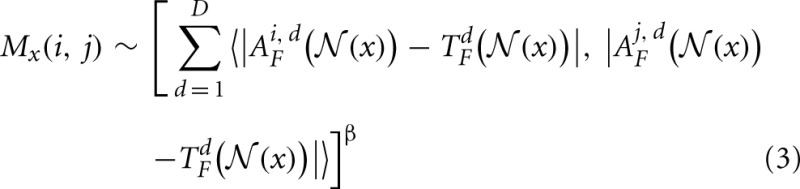



where *d* indexes through all imaging modality channels and *D* is the total number of imaging modalities. 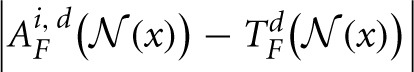 is the vector of absolute intensity difference between a warped atlas and the target image in the *d*_*th*_ modality channel over a local patch 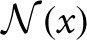 centered at *x* and 〈·,·〉 is the dot product. β is a model parameter. Note that if the off-diagonal elements in *M*_*x*_ are set to zeros, the voting weights derived from *M*_*x*_ is equivalent to the local weighted voting approach with the inverse distance weighting function as described in Artaechevarria et al. (2009). In this simplified case, β has a more straightforward interpretation that controls the distribution of voting weights. Large βs will produce more sparse voting weights and only the atlases that are most similar to the target image contribute to the consensus solution. Similarly, small βs will produce more uniform voting weights.

To make the measure more robust to image intensity scale variations across different images, we normalize each image intensity patch to have zero mean and a unit variance before estimating *M*_*x*_.

***2.1.1.3. The local search algorithm.*** To make label fusion more robust against registration errors, we apply a local search algorithm to find the patch from each warped atlas within a small neighborhood 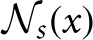 that is the most similar to the target patch in the target image. Under the assumption that more similar patches are more likely to be correct correspondences, instead of the original corresponding patches in the warped atlases, the searched patches are applied for label fusion.

We determine the *local search correspondence map* between the atlas *i* and the target image as follows:

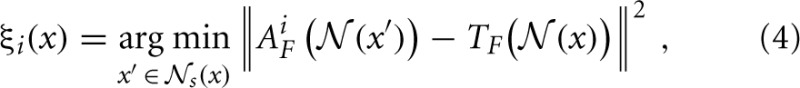



Note that the domain of the minimization above is restricted to a neighborhood 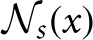. Given the set of local search correspondence maps {ξ_*i*_}, we refine the definition of the consensus segmentation as:

(5) $$\hat{p}\left(l|x,T_{F}\right)=\sum_{i=1}^{n}w^{i}(\xi_{i}(x))p\left(l|\xi_{i}(x),A^{i}\right),$$

The local search algorithm compares each target image patch with all patches within the searching neighborhood in each warped atlas. Normalizing image patches within the search neighborhood can be an expensive operation. To make the algorithm more efficient, we make the following observation. Let *X* and *Y* be vectors storing the original intensity values for two image patches. Let *x* and *y* be the normalized vector for *X* and *Y*, respectively. Let $\bar{Y}=\sum_{i=1}^{k}Y(i)/k$ and $\sigma (Y)={\left[\frac{\sum_{i=1}^{k}{\left(Y(i)-\bar{Y}\right)}^{2}}{k}\right]}^{0.5}$ be the mean and standard deviation for *Y*, where *k* is the vector size of *Y*. Hence, $y(i)=\frac{Y(i)-\bar{Y}}{\sigma (Y)}$. To compute the sum of squared distance between *x* and *y*, we have:

(6) $$\sum_{i=1}^{n}{\left[x(i)-y(i)\right]}^{2}=\sum_{i=1}^{n}{\left[x(i)-\frac{Y(i)-\bar{Y}}{\sigma (Y)}\right]}^{2}$$

(7) $$\begin{array}{l} \;=\frac{1}{\sigma {\left(Y\right)}^{2}}\sum_{i=1}^{k}[Y{\left(i\right)}^{2}-{\bar{Y}}^{2}+x{\left(i\right)}^{2}\sigma^{2}(Y)\\ \;-2x(i)Y(i)\sigma (Y)+2x(i)\bar{Y}\sigma (Y)] \end{array}$$

(8) $$\;=k+1-\frac{2}{\sigma (Y)}\sum_{i=1}^{k}x(i)Y(i)$$

Equation (8) is obtained from the fact that $\sum_{i=1}^{k}\left[Y{\left(i\right)}^{2}-{\bar{Y}}^{2}\right]=k\sigma {\left(Y\right)}^{2}$, $\sum_{i=1}^{k}x{\left(i\right)}^{2}=1$, and $\sum_{i=1}^{k}x(i)=0$. Hence, to make the local search algorithm more efficient, we only need to normalize the target image patch and search the patch in the warped atlas that minimizes $-\frac{1}{\sigma (Y)}\sum_{i=1}^{k}x(i)Y(i)$. Efficiency is achieved by avoiding the normalization operation for atlas patches during local search.

Note that, similar to the non-local mean patch based label fusion approach Coupe et al. (2011), employing all patches within the searching neighborhood for estimating the pairwise atlas dependencies produces more accurate estimation Wang et al. (2013a). However, this approach has much higher computational complexity. To make our label fusion software more practical, we choose the local search algorithm in our implementation.

***2.1.1.4. Parameter summary.*** The joint label fusion technique has four primary parameters:

- *r*_*p*_: the radius defining the image patch for estimating atlas dependencies (3).
- *r*_*s*_: the radius defining the search neighborhood 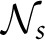.
- β: the model parameter for transferring image similarity measures into atlas dependencies in (3).
- α: the weight of the conditioning identity matrix added to *M*_*x*_.

***2.1.1.5. Joint label fusion user interface***. Our implementation, *jointfusion*, is based on Insight Toolkit (ITK), which allows us to take advantage of the image I/O functions implemented in ITK. *jointfusion* has the following user interface.


Joint Label Fusion
  usage:
  jointfusion dim mod [options] output_
  image
  required options:
    dim   Image dimension (2 or 3)
    mod   Number of imaging modalities
    -g    Warped atlas images
    -tg   Target image(s)
    -l    Warped atlas segmentations
    -m    <method> [parameters]
          Options: Joint[alpha,beta]
  other options:
    -rp   Appearance patch radius
    -rs   Local search radius
    -p    Output label posterior maps
          produced by label fusion


#### 2.1.2. Corrective learning

As we show in (Wang et al., 2011), automatic segmentation algorithms may produce systematic errors comparing to the gold standard manual segmentation. Such systematic errors could be produced due to the limitations of the segmentation model employed by the segmentation method or due to suboptimal solutions produced by the optimization algorithm. To reduce such systematic errors, corrective learning applies machine learning techniques to automatically detect and correct systematic errors produced by a “host” automatic segmentation method.

To illustrate how corrective learning works, we take a simple binary segmentation problem as an example. Using a set of example images, for which the gold standard manual segmentation is available, and to which the host method has been applied, we train a classifier [using AdaBoost (Freund and Schapire, 1997) in our current implementation] to discriminate between voxels correctly labeled by the host method and the voxels where the host method and the manual segmentation disagree. When segmenting a target image, the host method is first applied, and then each voxel is examined by the classifier. If the classifier believes that a voxel was mislabeled, its label is changed. In case of more than two labels, corrective learning needs to learn additional classifiers, as detailed below.

Note that machine learning is commonly used for image segmentation in computer vision (Kumar and Hebert, 2003; Tu and Bai, 2010) and medical image analysis (Tu et al., 2007; Morra et al., 2009; Tu and Bai, 2010). Typically, classifiers assigning labels to image voxels are trained and applied purely based on features extracted from images. By contrast, corrective learning allows the learning algorithm to benefit from the domain-specific knowledge captured by the host segmentation method. For instance, a host segmentation method may represent domain-specific knowledge in the form of shape priors and priors on spatial relations between anatomical structures. Corrective learning allows such high-level domain-specific knowledge to be incorporated into the learning process efficiently by using the segmentation results produced by the host method as an additional contextual feature (see more details below).

***2.1.2.1. Implementation***. In (Wang et al., 2011), we developed two corrective learning algorithms: explicit error correction (EEC) and implicit error correction (IEC). First, we define a working region of interest (ROI) to be derived from performing a dilation operation to the set of voxels assigned to non-background labels by the host method. Each voxel in the working ROI of each training image serves as a sample for training the corrective learning classifiers. The motivation for using the working ROI is that when the host method works reasonably well, most voxels labeled as foreground are in the close proximity of the foreground voxels in the manual segmentation. Hence, using a working ROI simplifies the learning problem by excluding most irrelevant background voxels from consideration.

In binary segmentation problems, IEC is equivalent to EEC. In a problem with *L* > 2 labels, EEC uses all voxels in the working ROI to train a single “error detection” classifier, whose task is to identify the voxels mislabeled by the host method. EEC then uses the voxels mislabeled by the host method to train *L* “error correction” classifiers, whose task is to reassign labels to the voxels identified as mislabeled by error detection. Each error correction classifier is designed to detect voxels that should be assigned each target label. To reassign labels to a voxel, it is evaluated by all *L* error correction classifiers, and the label whose classifier gives the highest response is chosen. By contrast, IEC treats all voxels within the working ROI as mislabeled and directly trains *N* error correction classifiers to reassign labels. In principle, EEC is more efficient than IEC for multi-label segmentation because IEC trains *N* error correction classifiers using all voxels in the working ROI, while EEC only uses a subset of voxels to train those correction classifiers. On the other hand, IEC has the advantage of not affected by incorrect error detection results.

To make corrective learning more efficient and more effective for segmentation problems with multiple labels, we implemented a third hybrid error correction strategy that combines the advantage of both EEC and IEC. This error correction strategy aims at problems with large numbers of labels by *incorporating the prior knowledge that when a host method works reasonably well, most voxels assigned by the host method to a foreground label are in the close proximity of the voxels manually assigned that label*. To improve the efficiency of IEC, we propose to restrict error correction for any foreground label only within the *label's working ROI*, derived by performing dilation to the set of all voxels assigned the label by the host method. To apply these trained classifiers to correct segmentation errors for a testing image, we apply each classifier to evaluate the confidence of assigning the corresponding label to each voxel within the label's working ROI. If a voxel belongs to the ROI of multiple labels, the label whose classifier gives the maximal response at the voxel is chosen for the voxel. Since error detection is not explicitly performed, our current implementation is simplified compared to the EEC algorithm. Furthermore, the implemented error correction strategy is not affected by incorrect error detection results. Compared with the IEC algorithm, our implementation is more efficient and more effective as only a small portion of the data, which are also more relevant to the problem of classifying the target label, are used to train the classifier for each label.

Note that the above label's working ROI definition has one limitation. If a host segmentation method fails to produce some segmentation labels, then the algorithm cannot recover the missing labels. To address this problem, we allow a second approach to define a label's working ROI by using a predefined ROI mask. If a ROI mask is provided for a label, the label's ROI is obtained from performing a dilation operation to the set of voxels in the mask. In principle, the ROI mask should cover most voxels of the target label. One way to define ROI masks for missing labels produced by the host method is to use the ROI of labels whose working ROIs cover most voxels manually assigned to the missing label. The union of these labels' working ROIs can be defined as the missing label's working ROI.

***2.1.2.2. Features***. Typical features that can be used to describe each voxel for the learning task include spatial, appearance, and contextual features. The spatial features are computed as the relative coordinate of each voxel to the ROI's center of mass. The appearance and contextual features are directly derived from the voxel's neighborhood image patch from the training image and the initial segmentation produced by the host method, respectively. To enhance the spatial correlation, the joint spatial-appearance and joint spatial-contextual features are also included by multiplying each spatial feature with each appearance and contextual feature, respectively. To include other feature types, one can compute features for each voxel and store the voxel-wise feature response into a feature image, i.e., the intensity at each voxel in the feature image is the feature value at that voxel. Passing these feature images to the algorithm, as shown below, will allow these features to be used in corrective learning.

Note that the above patch based features are not rotation or scale invariant. Hence, they are only suitable for images that have similar orientations and scales. Since many medical images, e.g., MRI and CT, are acquired under constrained rotations and scales, these features are often adequate in practice. For problems that do have large rotation and scale variations, one should apply more suitable features.

***2.1.2.3. Subsampling for large training dataset***. For large data set, it is not always possible to include all voxels within a label's working ROI for learning its classifier due to the memory constraint. For such cases, a subsampling strategy can be applied to randomly select a portion of training voxels according to a specified sampling percentage.

***2.1.2.4. Parameter summary***. The corrective learning technique has three primary parameters:

- *r*_*d*_: the radius for the dilation operation for defining each label's working ROI.
- *r*_*f*_: the radius defining the image patch for deriving voxel-wise features.
- *SampleRatio*: the portion of voxels within the label's working ROI to be used for learning the classifier for the label.

***2.1.2.5. Corrective learning user interface***. We separately implemented the algorithm for learning corrective classifiers and the algorithm applying these classifiers for making corrections. We name the program for learning corrective classifiers as *bl*, which stands for *bias learning* as it learns classifiers that capture the systematic errors, or bias, produced by an automatic segmentation algorithm. We name the program for making corrections as *sa*, which stands for *segmentation adapter* because it adapts the segmentation produced by the host method to be closer to the desired gold standard. These two programs have the following user interface.


Corrective Learning
  usage:
    bl dim [options] AdaBoost_Prefix
  required options:
    dim     Image dimension (2 or 3)
    -ms     Manual Segmentation
    -as     Automatic segmentation
    -tl     The target label
    -rd     Dilation radius
    -rf     Feature radius
    -rate   Training data sampling rate
    -i      Number of AdaBoost training
            iterations
  other options:
    -c      Number of feature channels
    -f      Feature images
    -m      ROI mask
Segmentation correction
  usage:
    sa input_segmentation AdaBoost_Prefix
       output_segmentation [options]
  options:
    -f      Feature images
    -p      Output posterior maps
    -m      ROI mask


### 2.2. Application 1: brain MRI segmention

To demonstrate the usage of the joint label fusion and corrective learning software, we provide implementation details for two applications: whole brain parcellation and canine leg muscle segmentation using MR images. In this section, we describe our application for brain segmentation. The software used in our experiments will be distributed through the Advanced Normalization Tools (ANTs) package Avants et al. (2008) and at http://www.nitrc.org/projects/picsl_malf.

#### 2.2.1. Data and manual segmentation

The dataset used in this study includes 35 brain MRI scans obtained from the OASIS project. The manual brain segmentations of these images were produced by Neuromorphometrics, Inc. (http://Neuromorphometrics.com/) using the brainCOLOR labeling protocol. The data were applied in the 2012 MICCAI Multi-Atlas Labeling Challenge and can be downloaded at (https://masi.vuse.vanderbilt.edu/workshop2012/index.php/Main_Page). In the challenge, 15 subjects were used as atlases and the remaining 20 images were used for testing.

#### 2.2.2. Image registration

To apply our algorithms, we need pairwise registered transformations between each atlas and each target image and between each pair of atlas images. To facilitate comparisons with other label fusion algorithms, we applied the standard transformations provided by the challenge organizers. For the brain image data, the standard transformations are produced by the ANTs registration tool and can be downloadable at http://placid.nlm.nih.gov/user/48. To generate warped images from the transformation files, we applied *antsApplyTransforms* with linear interpolation. To generate warped segmentations, we applied *antsApplyTransforms* with nearest neighbor interpolation.

#### 2.2.3. Joint label fusion

The following command demonstrates how to apply *jointfusion* to segment one target image, i.e., subject 1003_3.


./jointfusion 3 1 -g ./warped/*_to_1003_3_
                     image.nii.gz \
                  -l warped/*_to_1003_3_
                     seg_NN.nii.gz \
                  -m Joint[0.1,2] \
                  -rp 2x2x2 \
                  -rs 3x3x3 \
                  -tg ./Testing/1003_3.nii.
                      gz \
                  -p ./malf/1003_3_Joint_
                     posterior%04d.nii.gz \
                 ./malf/1003_3_Joint.nii.gz


In this application, only one MRI modality is available. Hence, mod = 1. The folder *warped* stores the warped atlases for each target image. We set the following parameters for *jointfusion*: α = 0.1, β = 2 and isotropic neighborhoods with radius two and three for *r*_*p*_ and *r*_*s*_, respectively. These parameters were chosen because they are optimal for segmenting the hippocampus in our previous study (Wang et al., 2013b). In addition to producing the consensus segmentation for the target subject, we also saved the posterior probabilities produced by label fusion for each anatomical label as images. These posterior images were applied as an additional feature for corrective learning, as described below. Note that we specify the file name of the output posterior images by the C *printf* format such that one unique posterior image is created for each label. For instance, for label 0 and 4, the generated posterior images are ./malf/1003_3_JointLabel_posterior0000.nii.gz and ./malf/1003_3_JointLabel_posterior0004.nii.gz, respectively.

To quantify the performance of *jointfusion* with respect to the four primary parameters, we also conducted the following leave-one-out cross-validation experiments using the training images. To test the impact of the appearance window size *r*_*p*_, we varied *r*_*p*_ from 1 to 3 and fixed *r*_*s*_ = 3, β = 2, β = 0.1. To test the impact of the local search window size, we varied *r*_*s*_ from 0 to 4 and fixed *r*_*p*_ = 2, β = 2, β = 0.1. We also varied β from 0.5 to 3 with a 0.5 step and fixed *r*_*p*_ = 2,*r*_*s*_ = 3, α = 0.1. Finally, we fixed *r*_*p*_ = 2,*r*_*s*_ = 3,β = 2 and tested with α = 0, 0.01, 0.05, 0.1, 0.2. For experiments testing the effects of *r*_*p*_ and *r*_*s*_, we report both computational time and segmentation accuracy for each parameter setting. Since varying β and α does not have significant impact on computational complexity, we only report segmentation accuracy for each parameter setting.

#### 2.2.4. Corrective learning

To apply corrective learning, we first applied joint label fusion with the above chosen parameters, i.e., (α, β, *r*_*p*_, *r*_*s*_,) = (0.1, 2, 2, 3), to segment each atlas image using the remaining atlases. With both manual segmentation and segmentation produced by joint label fusion, the atlases were applied for training the corrective learning classifiers. Recall that one classifier needs to be learned for each anatomical label. The following command trains the classifier for label 0, i.e., the background label.


./bl 3 -ms Training/*_glm.nii.gz \
       -as ./malf/1000_3_Joint.nii.gz \
           ...
           ./malf/1036_3_Joint.nii.gz \
       -tl 0 \
       -rd 1 \
       -i 500 \
       -rate 0.1 \
       -rf 2x2x2 \
       -c 2 \
       -f ./Training/1000_3.nii.gz \
          ./malf/1000_3_Joint_posterior
           0000.nii.gz \
          ...
          ./Training/1036_3.nii.gz \
          ./malf/1036_3_Joint_posterior
           0000.nii.gz \
       ./malf/BL/Joint_BL


We applied two feature images. In addition to the original intensity image, we also included the label posteriors generated by *jointfusion* for corrective learning. As we show in (Wang and Yushkevich, 2012), weighted voting based label fusion produces a spatial bias on the generated spatial label posteriors, which can be modeled as applying a spatial convolution on the ground truth label posteriors. Hence, the label posteriors produced by joint label fusion offers meaningful information for correcting such systematic errors. We set the dilation radius to be *r*_*d*_ = 1, which was shown to be optimal for correcting segmentation errors produced by multi-atlas label fusion for hippocampus segmentation in our previous study (Wang et al., 2011). For this learning task, a 10 percent sampling rate is applied.

We use the following command to apply the learned classifiers to correct segmentation errors for one testing image.


./sa ./malf/1003_3_Joint.nii.gz \
     ./malf/BL/Joint_BL \
     ./malf/1003_3_Joint_CL.nii.gz \
     -f ./Testing/1003_3.nii.gz ./malf/
       1003_3_Joint_posterior\%04d.nii.gz


Again, we used the C *printf* format to specify the file name of label posterior images as feature images.

Since we have shown in our previous work (Wang et al., 2011) that corrective learning is not sensitive to the dilation radius parameter. Here, we only conducted experiments to test the effect of the feature patch size *r*_*f*_ on the performance. We tested using *r*_*f*_ = 1 and *r*_*f*_ = 3 with the same dilation radius.

#### 2.2.5. Evaluation

To facilitate comparisons with other work, we follow the challenge evaluation criteria and evaluate our results using the Dice Similarity Coefficient (DSC) (Dice, 1945) between manual and automatic segmentation. DSC measures the ratio of the volume of overlap between two segmented regions and their average volume. For the brain image data, the results were evaluated based on 134 labels, including 36 subcortical labels and 98 cortical labels (see https://masi.vuse.vanderbilt.edu/workshop2012/index.php/Challenge_Details for details of the evaluation criterion). We separately report summarized results for all labels, cortical labels and subcortical labels. To give more information, we also report segmentation performances for nine subcortical structures, including accumbens area, amygdala, brain stem, caudate, cerebral white matter, CSF, hippocampus, putamen, and thalamus proper. For the canine lege data, evaluation iwas performed over all labels.

#### 2.2.6. Results

**Figure 1:**
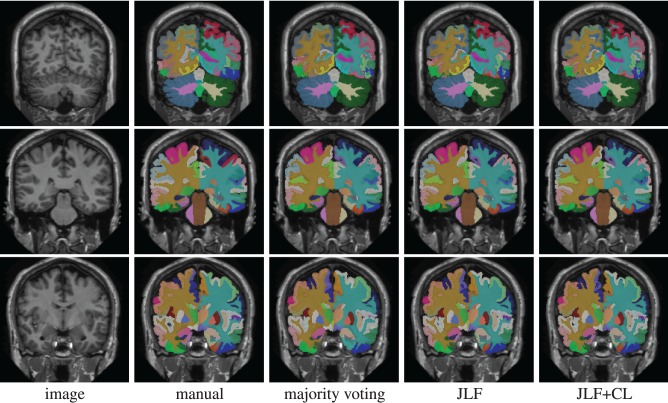
**Segmentations produced by manual segmentation, majority voting, joint label fusion (JLF), and joint label fusion combined with corrective learning (JLF+CL)**.

Using 15 atlases, *jointfusion* segments one image in about 1 h using a single core 2GHZ CPU with the parameter setting, *r*_*p*_ = 2, *r*_*s*_ = 3. Applying corrective learning to correct segmentation errors for an image can be done within a few minutes. Figure 1 shows some segmentation results produced by each method.

Table 1 reports the segmentation performance for majority voting, joint label fusion, and joint label fusion combined with correction learning. Joint label fusion produced an average DSC 0.757 for all labels, 0.732 for cortical labels, and 0.825 for subcortical labels. Corrective learning improved the results to 0.771, 0.747, and 0.836, respectively.

**Table 1:** **Segmentation performance in Dice Similarity Coefficient $\left(\frac{2|A\cap B|}{\left|A|+|B\right|}\right)$**.

**Anatomical region**	**Majority voting**	**Joint label fusion**	**Joint label fusion+ Corrective learning**
All labels	0.726 ± 0.138	0.757 ± 0.133	0.771 ± 0.131
Cortical labels	0.701 ± 0.113	0.732 ± 0.114	0.747 ± 0.115
Subcortical labels	0.796 ± 0.171	0.825 ± 0.154	0.836 ± 0.149
Left accumbens area	0.776 ± 0.074	0.804 ± 0.053	0.795 ± 0.048
Right accumbens area	0.759 ± 0.086	0.798 ± 0.058	0.795 ± 0.048
Left amygdala	0.800 ± 0.040	0.812 ± 0.032	0.815 ± 0.034
Right amygdala	0.808 ± 0.028	0.827 ± 0.025	0.830 ± 0.024
Brain stem	0.940 ± 0.009	0.943 ± 0.008	0.946 ± 0.008
Left caudate	0.801 ± 0.134	0.870 ± 0.101	0.881 ± 0.088
Right caudate	0.788 ± 0.122	0.865 ± 0.076	0.884 ± 0.070
Left cerebral white matter	0.903 ± 0.018	0.925 ± 0.019	0.937 ± 0.017
Right cerebral white matter	0.906 ± 0.018	0.926 ± 0.018	0.935 ± 0.019
CSF	0.723 ± 0.166	0.789 ± 0.092	0.820 ± 0.074
Left hippocampus	0.831 ± 0.046	0.862 ± 0.031	0.872 ± 0.023
Right hippocampus	0.830 ± 0.044	0.861 ± 0.027	0.871 ± 0.022
Left putamen	0.911 ± 0.029	0.915 ± 0.037	0.909 ± 0.042
Right putamen	0.909 ± 0.033	0.914 ± 0.040	0.907 ± 0.043
Left thalamus proper	0.903 ± 0.030	0.920 ± 0.014	0.921 ± 0.012
Right thalamus proper	0.903 ± 0.031	0.921 ± 0.012	0.923 ± 0.009

**Figure 2:**
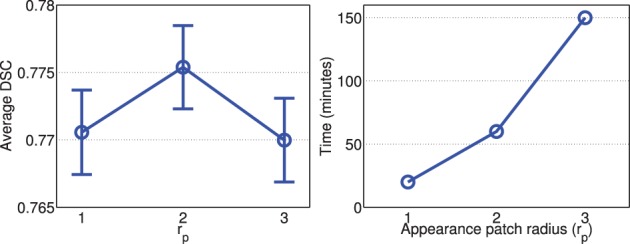
**Joint label fusion performance (Left: segmentation accuracy, error bars at ±0.05 standard deviation; Right: average processing time) with respect to image patch size**. Other parameters are set to *r*_*s*_ = 3, α = 0.1, β = 2.

**Figure 3:**
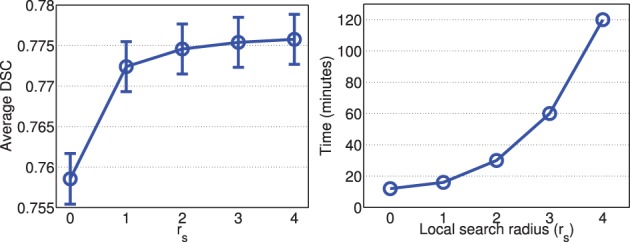
**Joint label fusion performance (Left: segmentation accuracy, error bars at ±0.05 standard deviation; Right: average processing time) with respect to local search neighborhood size**. Other parameters are set to *r*_*p*_ = 2, α = 0.1, β = 2.

Figures 2, 3 show the average processing time and average segmentation accuracy produced by joint label fusion with respect to *r*_*p*_ and *r*_*s*_, respectively. As expected, the processing time grows proportionally with respect to the neighborhood size. The performance of joint label fusion is not sensitive to the size of appearance patch *r*_*p*_, with the best performance produced by *r*_*p*_ = 2. In contrast, the local search algorithm produced more prominent improvement. Although applying larger searching neighbor consistently produced higher averaged DSC, applying *r*_*s*_ = 1 produced the greatest improvement. Further increasing *r*_*s*_ only slightly improved the segmentation accuracy.

**Figure 4:**
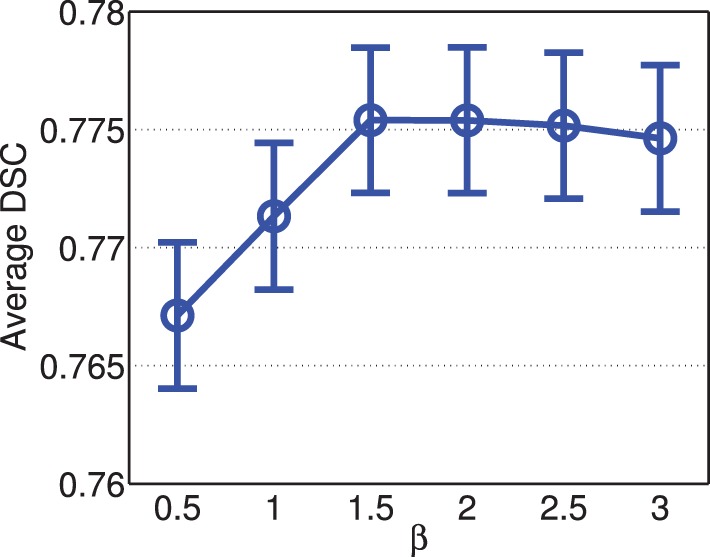
**Joint label fusion performance with respect to β (error bars at ±0.05 standard deviation)**. Other parameters are set to *r*_*p*_ = 2, *r*_*s*_ = 3, α = 0.1.

Figure 4 shows the segmentation accuracy produced by joint label fusion using different β values. For this application, the performance of joint label fusion is not sensitive to β. Among the tested β values, β = 1.5 produced the best segmentation accuracy.

**Figure 5:**
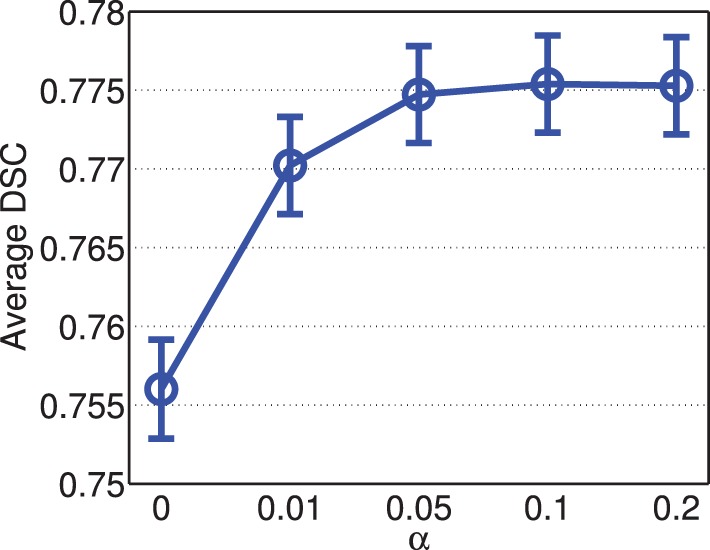
**Joint label fusion performance with respect to α (error bars at ±0.05 standard deviation)**. Other parameters are set to *r*_*p*_ = 2, *r*_*s*_ = 3, β = 2.

Figure 5 shows the segmentation accuracy produced by joint label fusion with respect to α. Adding the conditioning matrix, i.e., α>0, produced prominent improvement over without adding the conditioning matrix, i.e., α = 0. When the conditioning matrix is added, setting α between 0.01 and 0.2 has a slight impact on the performance, with the best performances achieved at α = 0.05 or 0.1.

**Figure 6:**
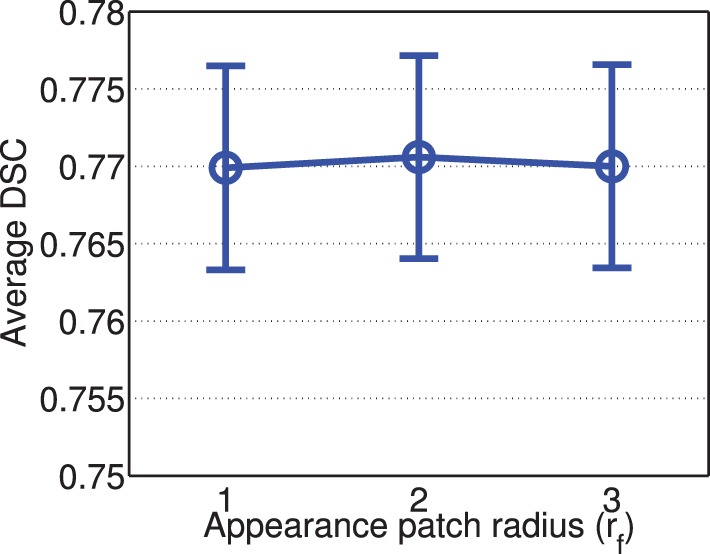
**Corrective learning performance with respect to image feature patch radius (error bars at ±0.05 standard deviation)**. Parameters for joint label fusion are set to *r*_*p*_ = 2, *r*_*s*_ = 3, α = 0.1, β = 2.

Figure 6 shows the segmentation performance produced by corrective learning with respect to feature patch radius. Again, we did not observe large performance variation. The performance produced by radius 2 is slightly better than those produced with radius 1 and 3.

### 2.3. Application 2: canine leg muscle segmentation

#### 2.3.1. Data and manual segmentation

The dataset used in this study contains 45 canine leg MR scans. For each dog, images were acquired with two MR modalities: a T2-weighted image sequence was acquired using a variable-flip-angle turbo spin echo (TSE) sequence and a T2-weighted fat-suppressed images (T2FS) sequence was then acquired using the same variable-flip-angle TSE sequence with the same scanning parameters except that a fat saturation preparation was applied. Seven proximal pelvic limb muscles were manually segmented: cranial sartorius, rectus femoris, semitendinosus, biceps femoris, gracilis, vastus lateralis and adductor magnus. In the challenge, 22 subjects were used as atlases and the remaining 23 subjects were used for testing. We will use this dataset for validating the multi-modality extension to our joint label fusion algorithm.

#### 2.3.2. Image registration

For this challenge, we produced the standard registration using ANTs, which can be downloaded at https://masi.vuse.vanderbilt.edu/workshop2013/index.php/Segmentation_Challenge_Details. Avants et al. (2013) contains details for how the registrations were generated. To quantify the accuracy of the standard transformations, we applied majority voting to generate a baseline segmentation performance.

#### 2.3.3. Joint label fusion

The following command demonstrates how to apply *jointfusion* to segment one target image, i.e., subject DD_039, using both MR modality channels.


./jointfusion 3 2 -g ./canine-lege-warped/
                     DD_040_to_DD_039_T2.
                     nii.gz \
                     ./canine-lege-warped/
                     DD_040_to_DD_039_T2FS.
                     nii.gz ... \
                  -l warped/*_to_DD_039_seg.
                     nii.gz \
                  -m Joint[0.1,0.5] \
                  -rp 2x2x2 \
                -rs 3x3x3 \
                -tg ./canine-legs/testing-
                    images/DD_039_T2.
                    nii.gz \
                    ./canine-legs/testing-
                    images/DD_039_T2FS.
                    nii.gz \
                -p. /canine-legs-malf/DD_
                    039_Joint_posterior%04d.
                    nii.gz \
                ./canine-legs-malf/DD_039_
                Joint.nii.gz


Note that, for this application, we applied β = 0.5. This parameter was chosen because it produced the optimal results for the cross-validation experiments on the training images as described below. To compare with the performance produced by using a single modality and by using two modalities, we also applied *jointfusion* by only using the T2-weighted image.

Since *r*_*s*_ and β have the most impact on the joint label fusion performance, for this application we only conducted experiments to quantify the performance of *jointfusion* with respect to these two parameters using leave-one-out cross-validation experiments on the training images. We varied σ from 0.5 to 2.5 with a 0.5 step while fixing *r*_*p*_ = 2,*r*_*s*_ = 3, λ = 0.1. We varied *r*_*s*_ from 0 to 4 while fixing *r*_*p*_ = 2, β = 0.5, λ = 0.1.

#### 2.3.4. Corrective learning

To apply corrective learning, again we first applied joint label fusion with the above chosen parameters, i.e., (α, β, *r*_*p*_, *r*_*s*_,) = (0.1,0.5,2,3), to segment each atlas image using the remaining atlases. With both manual segmentation and segmentation produced by joint label fusion, the atlases were applied for training the corrective learning classifiers. The following command trains the classifier for the background label.


./bl 3 -ms ./canine-legs/training-labels/*_
           seg.nii.gz \
       -as ./canine-legs-malf/DD_040_Joint.
           nii.gz \
           ...
           ./canine-legs-malf/DD_173_Joint.
           nii.gz \
       -tl 0 \
       -rd 1 \
       -i 500 \
       -rate 0.05 \
       -rf 2x2x2 \
       -c 3 \
       -f ./canine-legs/training-images/DD_
          040_T2.nii.gz \
          ./canine-legs/training-images/DD_
          040_T2FS.nii.gz \
          ./canine-legs-malf/DD_040_Joint_
          posterior0000.nii.gz \
          ...
          ./canine-legs/training-images/DD_
          173_T2.nii.gz \
          ./canine-legs/training-images/DD_
          173_T2FS.nii.gz \
          ./canine-legs-malf/DD_173_Joint_
          posterior0000.nii.gz \
       ./canine-legs-malf/BL/Joint_BL


We use the following command to apply the learned classifiers to correct segmentation errors for one testing image.


./sa ./canine-legs-malf/DD_039_Joint.
     nii.gz \
     ./canine-legs-malf/BL/Joint_BL \
     ./canine-legs-malf/DD_039_Joint_CL.
     nii.gz \
     -f ./canine-legs/testing-images/DD_
        039_T2.nii.gz \
        ./canine-legs/testing-images/DD_
        039_T2FS.nii.gz \
        ./canine-legs-malf/DD_039_Joint_
        posterior\%04d.nii.gz


#### 2.3.5. Results

**Figure 7:**
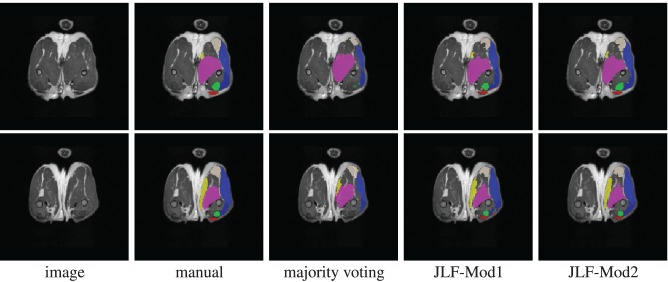
**Segmentations produced by manual segmentation, majority voting, joint label fusion with one imaging modality (JLF-Mod1), and joint label fusion with two imaging modalities (JLF-Mod2)**.

Using 22 atlases and both imaging modalities, *jointfusion* segments one image in about 1 h using a single core 2GHZ CPU with the parameter setting, *r*_*p*_ = 2, *r*_*s*_ = 3. Applying corrective learning to correct segmentation errors for an image can be done within 1 min. Figure 7 shows some segmentation results produced by each method.

**Figure 8:**
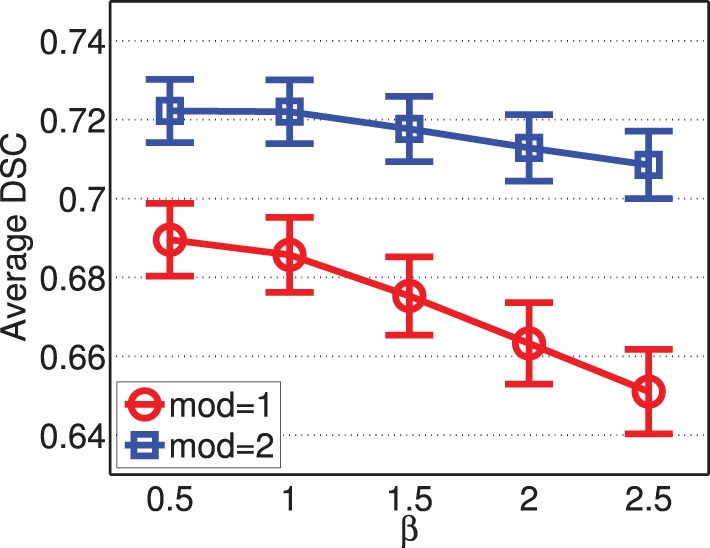
**Joint label fusion performance with respect to β (error bars at ±0.05 standard deviation)**. Other parameters are set to *r*_*p*_ = 2, *r*_*s*_ = 3, α = 0.1.

Figure 8 shows the segmentation accuracy produced by joint label fusion using different β values. The results produced by using a single modality and by using two modalities are given separately. As expected, multi-modality based label fusion did result in substantial performance improvement over using a single modality. For this application, the performance of joint label fusion is more sensitive to β when only one modality is applied. Among the tested β values, β = 0.5 produced the best segmentation accuracy.

**Figure 9:**
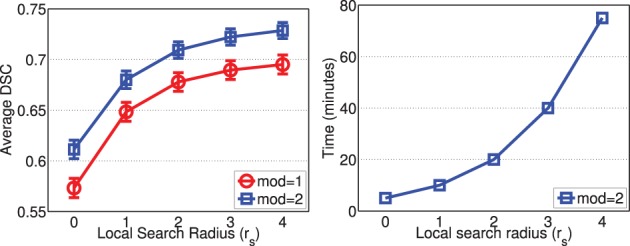
**Joint label fusion performance (Left: segmentation accuracy, error bars at ±0.05 standard deviation; Right: average processing time) with respect to local search neighborhood size**. Other parameters are set to *r*_*p*_ = 2, α = 0.1, β = 0.5.

Figure 9 shows the segmentation accuracy produced by joint label fusion with respect to *r*_*s*_. Since image registrations for canine leg images have lower quality than those produced for brain images, the local search algorithm produced more substantial improvement for this application than for brain segmentation. The average processing time produced by joint label fusion using two modalities is also given in Figure 9.

Table 2 reports the segmentation performance produced by majority voting, joint label fusion using a single imaging modality and joint label fusion using two imaging modalities from the leave-one-out cross-validation experiment on the training dataset. Table 3 reports the segmentation performance generated by the challenge organizer during the challenge competition produced by majority voting and joint label fusion combined with corrective learning.

**Table 2:** **Segmentation performance in Dice Similarity Coefficient $\left(\frac{2|A\cap B|}{\left|A|+|B\right|}\right)$ produce by leave-one-out cross validation using the canine leg muscle training data**.

**Anatomical region**	**Majority voting**	**Joint label fusion mod1**	**Joint label fusion mod2**
All labels	0.411 ± 0.274	0.690 ± 0.185	0.722 ± 0.160

**Table 3:** **Segmentation performance in Dice Similarity Coefficient $\left(\frac{2|A\cap B|}{\left|A|+|B\right|}\right)$ produce for the canine leg muscle testing data**.

**Anatomical region**	**Majority voting**	**Joint label fusion + corrective learning**
All Labels	0.418 ± 0.108	0.762 ± 0.098

## 3. Discussion

### 3.1. Comparison to the state of the art

Our algorithms participated in the MICCAI 2012 and 2013 multi-atlas labeling challenge competition. Our results on the canine leg muscle dataset are the best among all 2013 challenge entries for this dataset (see Asman et al., 2013 for more detail). Our results for brain segmentation produced based on the standard registration transforms are better than what we originally produced during the competition (Landman and Warfield, 2012). In the 2012 challenge, applying joint label fusion alone, our results are 0.750 for all labels, 0.722 for cortical labels, and 0.827 for subcortical labels. Combining joint label fusion and corrective learning, we produced the best results in the challenge competition, with 0.765 for all labels, 0.739 for cortical labels, and 0.838 for subcortical labels. In this study, applying joint label fusion alone, our results are 0.757 for all labels, 0.732 for cortical labels, and 0.825 for subcortical labels. Combining joint label fusion with corrective learning, our results are 0.771 for all labels, 0.747 for cortical labels, and 0.836 for subcortical labels. Note that, most improvements in our current study are for cortical labels. Hence, it is reasonable to expect that the standard registration transforms provided by the challenge organizers have better accuracy for the cortical regions than those produced by us during the challenge competition.

### 3.2. Parameter selection

We found that both joint label fusion and corrective learning are not sensitive to the parameter setting in this brain MRI segmentation application. However, using large local appearance neighborhood, e.g., *r*_*p*_ > 2, and large local search neighborhood, *r*_*s*_ > 2, significantly increase the computational cost. Hence, when computational cost is a limiting factor, one could achieve a good trade off between computational complexity and segmentation performance by choosing proper values for these two parameters. Based on our experiments, setting *r*_*p*_ = 1, 2 and *r*_*s*_ = 1, 2 can produce almost optimal performance and keep joint label fusion using 15 atlases within 30 min for whole brain segmentation.

For α, the weight for adding the conditioning matrix, we found that adding conditioning matrix is important for joint label fusion. To make sure that the added conditioning matrix is sufficient to avoid inverting an ill-conditioned matrix and the resulting voting weights also give a solution close to the global minimum of the original objective function, α should be chosen with respect to the scale of the estimated dependency matrix *M*_*x*_. According to our experiments, we found that setting α ≃ 1% of the scale of estimated *M*_*x*_ seems to be a good choice.

For the model parameter β used in estimating appearance based pairwise atlas dependencies Equation (8), its selection depends on the registration quality produced for the application at hand. Based on our experiments and our previous study (Wang et al., 2013b), we found that when registration can be done in good quality such as brain MRI registration in this study, setting β ≃ 2 is optimal. For mitral valve segmentation in ultra sound images (Wang et al., 2013a) and canine leg muscle segmentation, where good image registration is more difficult to produce due to low image quality and greater deformations, we found that setting β = 1 or 0.5 is optimal. Hence, setting β depends more on the application.

As we have applied in paper, one way to determine the optimal parameter settings is based on a leave one out experiment on the atlas set. That is segmenting each atlas using the remaining atlases with different parameter settings, the setting produced the best overall segmentation for all atlases should be chosen. As training classifiers in corrective learning, parameter selection for joint label fusion can be done offline. Hence, no additional burden is added for online label fusion. Similarly, combining corrective learning with multi-atlas label fusion is a natural choice, as no additional training data is need for corrective learning and no significant additional online computational burden is added by applying corrective learning.

### 3.3. Future work

Note that when the host segmentation method produces more accurate solutions, applying corrective learning further improves the overall accuracy. Hence, efforts on improving label fusion and corrective learning can be conducted in parallel. For improving corrective learning, one direction would be to explore more effective features and more effective learning algorithms. As recent studies (Montillo et al., 2011; Zikic et al., 2012) have shown that random forrest (Breiman, 2001) is a highly effective learning algorithm for addressing segmentation problems. Hence, replacing AdaBoost with random forrest may result in further improvement.

### Conflict of interest statement

The authors declare that the research was conducted in the absence of any commercial or financial relationships that could be construed as a potential conflict of interest.
